# Performance of AI vs radiology residents in the detection of intracranial hemorrhage on emergency CT: a real-world analysis

**DOI:** 10.1007/s00330-026-12385-y

**Published:** 2026-02-21

**Authors:** Quentin Pedrini, Periktioni Crina Chrysostomou, Pierre-Alexandre Poletti, Minerva Becker, Thomas Perneger, Rodolphe Meyer, Alexandra Platon

**Affiliations:** 1 Division of Radiology, Diagnostic Department, Geneva University Hospitals, Geneva, Switzerland; 2 Faculty of Medicine, University of Geneva, Geneva, Switzerland; 3 Department of Information Systems, Geneva University Hospitals, Geneva, Switzerland

**Keywords:** Tomography (X-ray computed), Intracranial hemorrhages, Emergencies, Artificial intelligence

## Abstract

**Objective:**

To evaluate the performance of a commercial artificial intelligence (AI) software in detecting intracranial hemorrhage (ICH) in emergency settings, compared to on-call radiology residents.

**Materials and methods:**

All consecutive unenhanced cerebral CT-scans performed in a single center over a 3-month period in the emergency department in patients with suspected ICH, initially interpreted by radiology residents on-call and subsequently verified and approved by a board-certified radiologist, were concomitantly analyzed by an AI software for the presence of ICH. Results from the AI software were stored in a separate PACS partition and were unavailable to the radiologists for the case reading.

We assessed the diagnostic performance of the AI software and of the radiology residents in detecting ICH. The reference standard was the final report of the board-certified radiologist.

**Results:**

Radiology reports of 2153 CT-scans were analyzed, and ICH prevalence was 15.4% (331/2153). The AI software achieved an overall sensitivity of 84% and a specificity of 94.4%, and radiology residents achieved a sensitivity of 96.4% and a specificity of 99.6%, respectively (*p*-values < 0.001). The sensitivity was 97.7% for AI and 98.5% for residents when CT examinations displayed an association of multiple hemorrhagic types (*p* = 1). The sensitivity was 95.2% for AI and 98.4% for radiology residents in the presence of multiple ICH sites (*p* = 0.11).

**Conclusion:**

Radiology residents demonstrated a significantly higher performance in detecting ICH compared to the AI software. AI exhibited very good diagnostic performance only in the presence of multiple hemorrhagic sites or multiple hemorrhage types.

**Key Points:**

***Question*** *How does the performance of the AI software compare to that of radiology residents in detecting ICH on unenhanced CT in real-life emergency workflow conditions?*

***Findings*** *In the emergency setting, the AI software demonstrated lower overall sensitivity and specificity than radiology residents for detecting ICH*.

***Clinical relevance*** *In real-life emergency conditions at a university hospital, the AI software did not offer a superior performance compared to radiology residents in detecting ICH. The integration of AI in this specific setting remains to be defined*.

**Graphical Abstract:**

**Figure:**
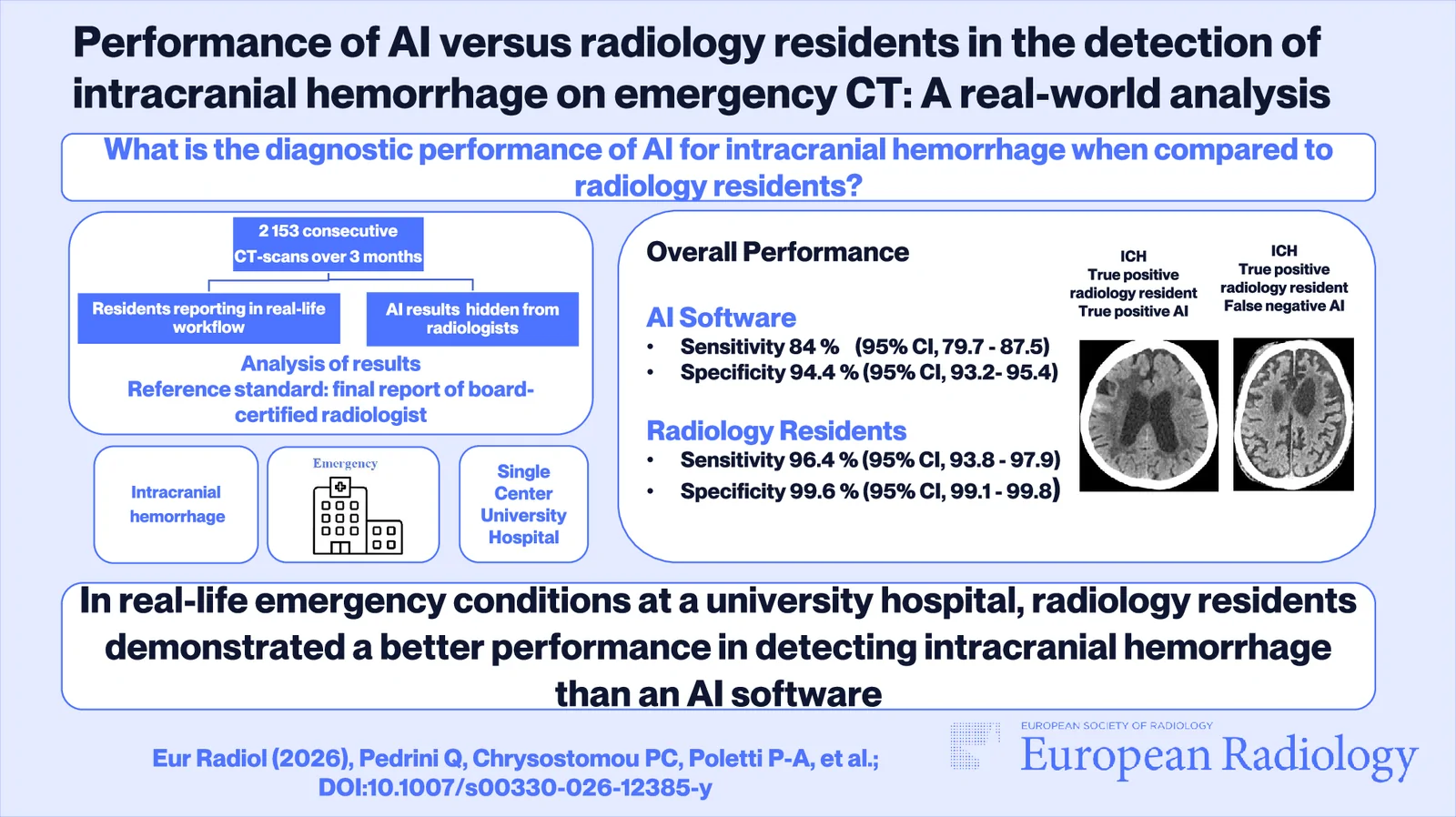

## Introduction

Intracranial hemorrhage (ICH), whether posttraumatic or secondary to various neurological causes, constitutes a true medical emergency and accounts for a significant proportion of emergency consultations [1, 2]. Unenhanced computed tomography (CT) is the standard imaging modality in acute settings for detecting ICH, due to its rapidity, high sensitivity, and accessibility, enabling prompt diagnosis and intervention that are crucial for improving patients’ outcomes [3, 4].

In recent years, artificial intelligence (AI) has garnered significant interest for detecting ICH on non-contrast CT scans. Numerous studies reported high sensitivity and specificity of various AI algorithms [5–7]. However, despite these promising preliminary results, the introduction of AI software in clinical practice is still hampered by various challenges, such as validation of the AI models on various clinical datasets or the lack of generalizability due to different validation methodologies [8]. Moreover, most studies have focused on technical performances, rather than evaluating AI in real-world clinical settings, particularly in comparison with radiology residents in emergency contexts [8].

The aim of our study was to evaluate the performance of a commercial AI tool in detecting ICH on unenhanced emergency CT scans, and to compare it with the diagnostic performance of radiology residents who provided immediate preliminary interpretations as part of the routine emergency workflow in a university hospital setting.

## Materials and methods

### Settings

Our single-center study received the IRB approval (IRB 2020-02812), which waived patients’ informed consent. The study was prospectively performed over a period of 3-months in the emergency radiology section of the Emergency Department (ED) of the Geneva University Hospitals; analysis was performed retrospectively, based on radiology reports and AI results. The emergency radiology section operates with two CT-scanners and provides round-the-clock imaging for all traumatic and non-traumatic conditions, both neurological and body pathologies. A radiology resident is present 24/7, overseeing the CT activity and delivering the preliminary report for each CT examination. These reports are verified by the attending board-certified radiologists, and once validated, they become the final interpretation for the CT examination. In our department, residents begin working in the emergency radiology section at the end of their 2nd year of postgraduate training, after completing their mandatory rotations in body and neuro CT, sonography, and conventional radiology.

### Study population and design

**Fig. 1:**
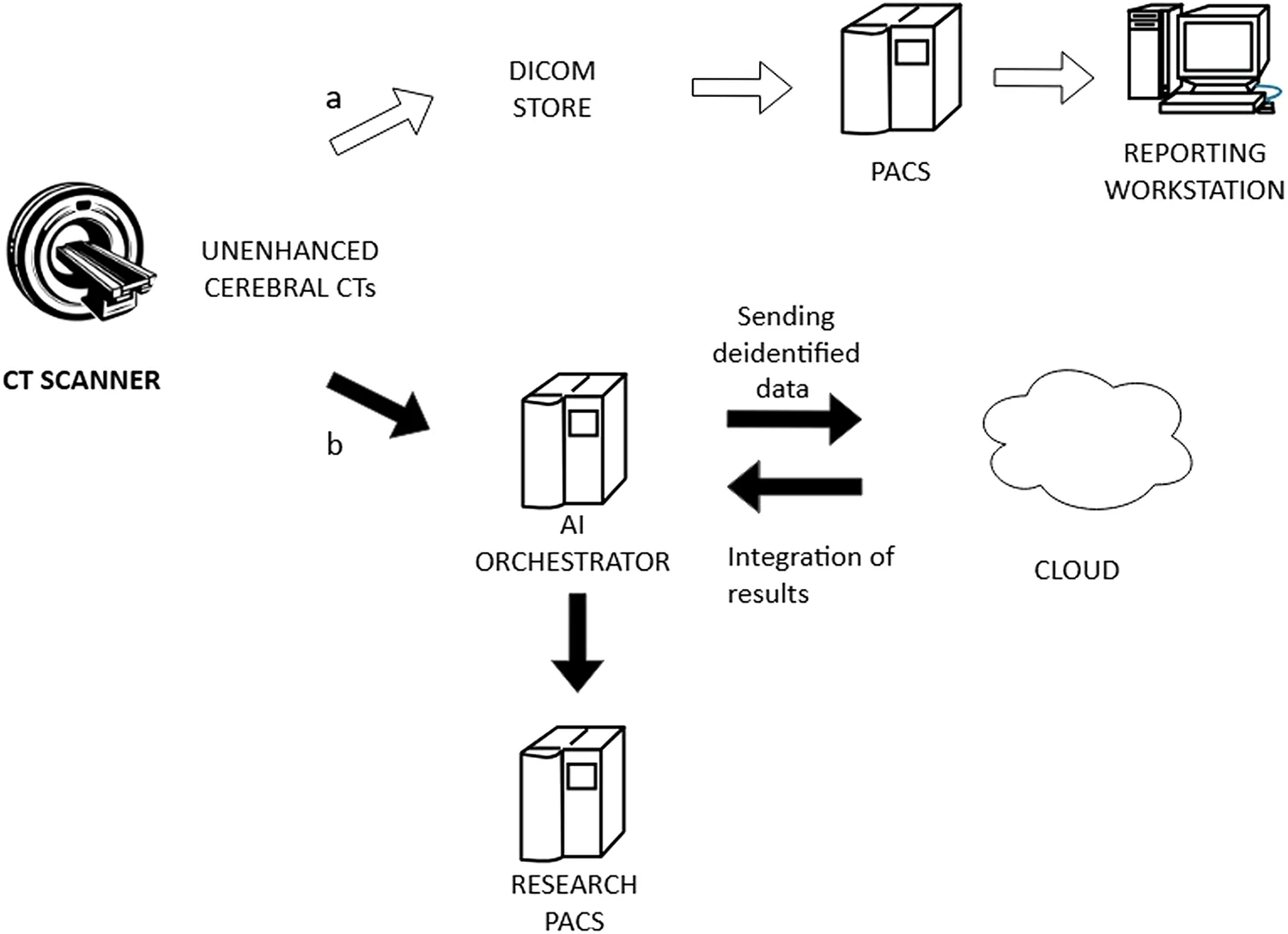
Routine interpretation and AI workflow for unenhanced cerebral CT scans during the study period. White arrows (**a**): routine interpretation workflow. Black arrows (**b**): parallel workflow for AI, separated and independent from the routine interpretation workflow

We prospectively included all consecutive unenhanced cerebral CT examinations of adult patients (≥ 16 years old), performed in the emergency setting, for suspected ICH, either as a primary clinical indication or as a differential diagnosis. We excluded from analysis the CT examinations with reports signed only by board-certified radiologists. During the study period, we implemented a separate informatics workflow, independent of the standard CT interpretation system, to allow the direct transfer of unenhanced cerebral CT examinations from the CT console to a commercially available software, trained for the automatic detection of ICH. The results of the AI analysis were sent to a dedicated picture archiving and communication system (PACS) partition (research PACS) and were not accessible to residents or attending radiologists during the interpretation of the CT examinations. Both radiology residents and attending radiologists were blinded to the study objectives and to the AI results. The workflow implemented during the study period is shown in Fig. 1.

### CT acquisition

All unenhanced cerebral CT examinations were performed on one of the two CT scanners in the emergency radiology section (256-multi-slices Somatom Force and 128-multi-slices Somatom Definition Edge, Siemens Healthineers). Unenhanced CT scans were performed in spiral mode, with a pitch of 0.55, 128 × 0.6 mm collimation, and 3/1 mm image reconstruction, using an Hf38s/3 kernel. On Somatom Force, automatic tube current modulation (CARE Dose4D) and semi-automatic tube voltage selection (120 kV reference) were employed. On Somatom Edge, scans were performed at a fixed 120 kV, with tube current modulation (X-CARE). The same reconstructed series was sent for routine clinical reading and for AI analysis.

### AI software

The evaluation of the software for the detection of ICH was conducted as part of a specific research protocol and not within the framework of its commercial use. In its commercial use, the AI software is a radiological computer-assisted triage and notification tool intended to assist radiologists with workflow triage by flagging and communicating suspected positive findings of ICH [9, 10]. Within the frame of our research, the case notification to the radiologists and the list-prioritization features were not deployed. For this study, we have used the commercially available software Aidoc (V 1.0). The software labeled the CT images considered positive for ICH using a heatmap. These labeled images were sent directly to a research PACS and thus not available to radiology residents nor to attending radiologists for review during interpretation of CT examinations; the research PACS was protected by specific access rights.

The software results, presented in binary mode (presence or absence of ICH), were kept by an informatics analyst not involved in the study, and released to the principal investigator (AP) six months after data collection.

### Collected data

Clinical and radiological data were retrospectively collected from the radiological information system of the Radiology Division of our hospital. For all included examinations, we retrieved the patients’ sex, age, indication for CT, presence of ICH in the residents ‘preliminary reports, and in the final reports of the attending radiologists. A visual check of the AI-labeled images was done by an attending radiologist, with more than 20 years’ experience in emergency radiology and specific training in neuroradiology.

Based on the ED physician prescription, we further categorized the primary indication for the CT examination into three groups: CT performed for suspected ICH (traumatic or non-traumatic) as the primary indication; CT performed for suspected cerebral ischemia (including potential ICH), and CT performed for suspicion of other cerebral pathologies, with ICH as a differential diagnosis.

The type of ICH was categorized according to its location as intraparenchymal, subarachnoid, epidural, or subdural; furthermore, subdural hematomas (SDH) were classified into acute, subacute, and chronic subtypes. We considered ICH as an association of multiple types of hemorrhage when these were present concomitantly on the same CT examination. Furthermore, ICH was categorized as single site vs multiple sites if, for the latter, numerous foci of ICH were present either in supra and infratentorial locations or bilaterally regarding the midline.

### Reference standard

The reference standard for the presence or absence of ICH was the final CT report of the attending radiologist. Both the preliminary reports signed by the residents and the results provided by the software were compared against this standard.

### Statistics

Analyses were performed using IBM SPSS Statistics version 25 (IBM©). We considered the diagnosis of presence or absence of ICH made by the attending radiologists as the reference standard. The diagnoses made by the AI software and the residents were treated as the “diagnostic tests” to be evaluated. For each “diagnostic test” we obtained sensitivity and specificity (vs the attending radiologist), as well as positive and negative predictive values. Exact 95% confidence intervals (95% CIs) were computed using the Wilson method [11]. We compared the values of sensitivity (among true positives) and specificity (among true negatives) between subgroups, using Fisher's exact test for independent groups and between methods (AI software vs residents) within subgroups using the McNemar chi-square test for paired data. Significance levels were set for all comparisons at *p* < 0.05.

## Results

We included 2158 consecutive unenhanced CT-scans performed in the ED during the study period. A diagnosis by the AI software was available for all 2158 cases, but the resident’s diagnosis was missing for 5 of them; thus, 2153 CT-scans remained for the analysis.

Among the 2153 included examinations, 48.3% (1 039) were women, mean age was 62.3 years. Patients’ characteristics, by age, sex, indication for CT, and presence of ICH, are shown in Table 1.

**Table 1:** Characteristics of 2153 patients who had CT-scans performed for the diagnosis of ICH in the emergency room, and prevalence of confirmed hemorrhage

	*N* (%)	ICH (%)	*p*-value
All	2153 (100)	331 (15.4%)	
Gender			0.002
Women	1039 (48.3)	12.8%	
Men	1114 (51.7)	17.6%	
Age			0.13
16–49	645 (30.0)	15.8%	
50–69	540 (25.1)	17.6%	
70–102	968 (44.9)	13.8%	
Indication			< 0.001
Suspected hemorrhage	1235 (57.4)	20.1%	
Suspected ischemia	761 (35.3)	8.9%	
Suspected various intracranial pathologies	157 (7.3)	9.6%	

*ICH* intracranial hemorrhage

Overall, 331 cases received a diagnosis of ICH from the attending radiologist, the prevalence was 15.4%. ICH was more frequent among men (17.6%) than among women (12.8%), and more frequent when the clinical indication for the CT-scan was suspected hemorrhage (20.1%) rather than ischemia (8.9%) or other suspected intracerebral pathologies (9.6%). Among the ICH, association of multiple types of ICH was present in 130 (39.3%) of cases, subarachnoid hemorrhage in 70 (21.1%), SDH in 65 (19.6%), intraparenchymal hemorrhage in 58 (17.5%), and epidural hematoma in 8 (2.4%) cases.

### Diagnostic performance analysis

Overall, the AI software had a good sensitivity: 84% (95% CI: 79.7–87.5) and positive predictive value: 73.2% (95% CI: 68.5–77.4), and an excellent specificity: 94.4% (95% CI: 93.2–95.4) and negative predictive value: 97% (95% CI: 96.1–97.7). However, all four performance measures were substantially better for radiology residents, sensitivity: 96.4% (95% CI: 93.8–97.9), *p* < 0.001, positive predictive value: 97.6% (95% CI: 95.2–98.8), specificity: 99.6% (95% CI: 99.1–99.8), *p* < 0.001 negative predictive value: 99.3% (95% CI: 98.9–99.6) respectively than for the AI software (Table 2).

**Table 2:** Global performance of the AI software and of radiology residents for the diagnosis of ICH, in comparison with the diagnosis of the attending radiologist (reference standard), in 2 153 CT-scans

	AI software (%, 95% CI)	Radiology residents (%, 95% CI)	Between methods *p*-value
Sensitivity (%)	84.0 (79.7–87.5)	96.4 (93.8–97.9)	< 0.001
Specificity (%)	94.4 (93.2–95.4)	99.6 (99.1–99.8)	< 0.001
Positive predictive value (%)	73.2 (68.5–77.4)	97.6 (95.2–98.8)	
Negative predictive value (%)	97.0 (96.1–97.7)	99.3 (98.9–99.6)	

Point estimates are percentages, 95% confidence intervals are in parentheses

*ICH* intracranial hemorrhage

These results held across different indications for CT. The sensitivity of the AI software was lower when the indication for CT was hemorrhage (80.6%) rather than ischemia (94.1%) or other intracranial pathologies (93.3%), but specificity remained similar. The performance of the radiology residents was stable and very good across all subgroups of indications for CT (96.4% for suspected hemorrhage, 97.1% for suspected ischemia, and 93.3% for suspected other intracranial pathologies, respectively). The between-method comparisons favored the residents in all situations except for sensitivity in patients with a suspicion of ischemia or other intracranial pathology, where the results were similar for residents and the AI software (Table 3).

**Table 3:** Sensitivity and specificity of the AI software and of radiology residents across subgroups of patients with suspected ICH

	Sensitivity			Specificity		
	AI software (%)	Radiology residents (%)	Between-methods *p*-value	AI software (%)	Radiology residents (%)	Between-methods *p*-value
Overall	84.0	96.4	< 0.001	94.4	99.6	< 0.001
Gender	(*p* = 0.24)	(*p* = 0.075)		(*p* = 0.26)	(*p* = 0.50)	
Women	82.0	94.0	0.002	95.0	99.4	< 0.001
Men	86.7	97.9	< 0.001	93.8	99.7	< 0.001
Age	(*p* = 0.09)	(*p* = 0.88)		(*p* = 0.17)	(*p* = 0.40)	
16–49	88.2	96.1	0.039	95.9	99.3	< 0.001
50–69	87.4	95.8	0.039	93.7	99.6	< 0.001
70–102	78.9	97.0	< 0.001	93.8	99.8	< 0.001
Indication	(*p* = 0.016)	(*p* = 0.78)		(*p* = 0.31)	(*p* = 0.08)	
Hemorrhage	80.6	96.4	< 0.001	94.6	99.3	< 0.001
Ischemia	94.1	97.1	0.69	94.7	100	< 0.001
Various intracranial pathologies	93.3	93.3	1.00	91.5	99.3	0.003

Between-subgroups *p*-values are in parentheses

*ICH* intracranial hemorrhage

**Fig. 2:**
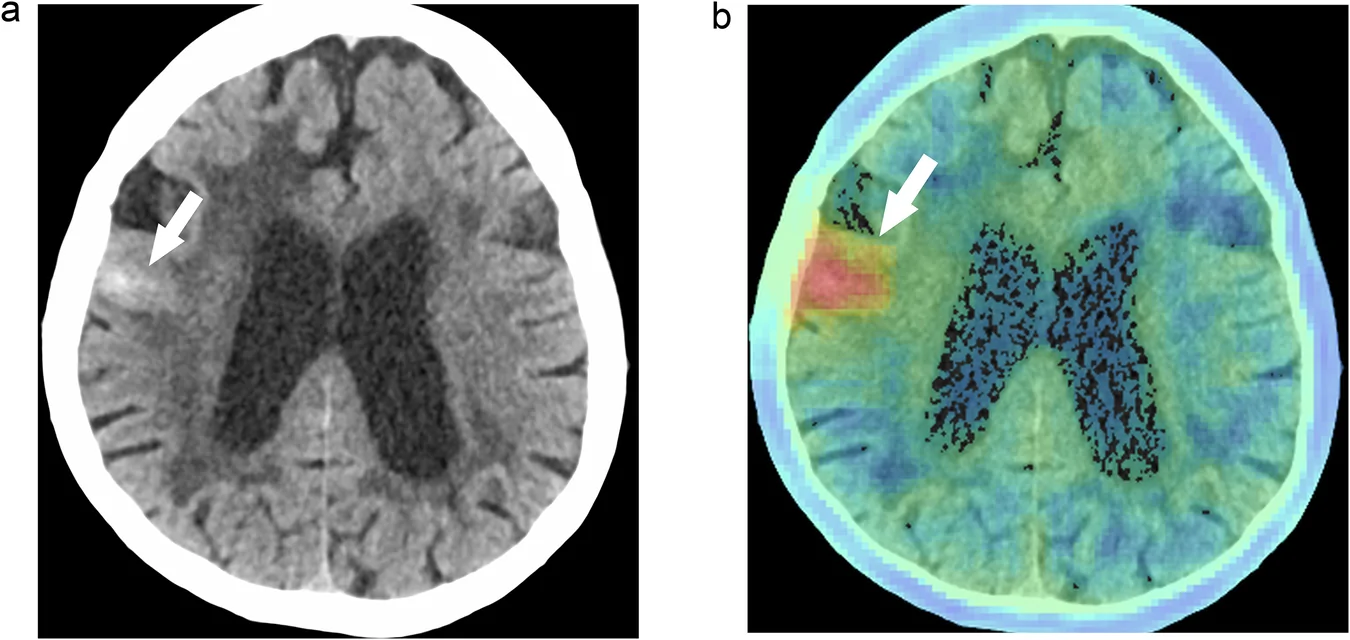
ICH, true-positive for the AI software. **a** Unenhanced cerebral CT: right parietal subarachnoid hemorrhage (arrow). **b** The subarachnoid hemorrhage is correctly color-labeled by AI on the heatmap (arrow)

**Fig. 3:**
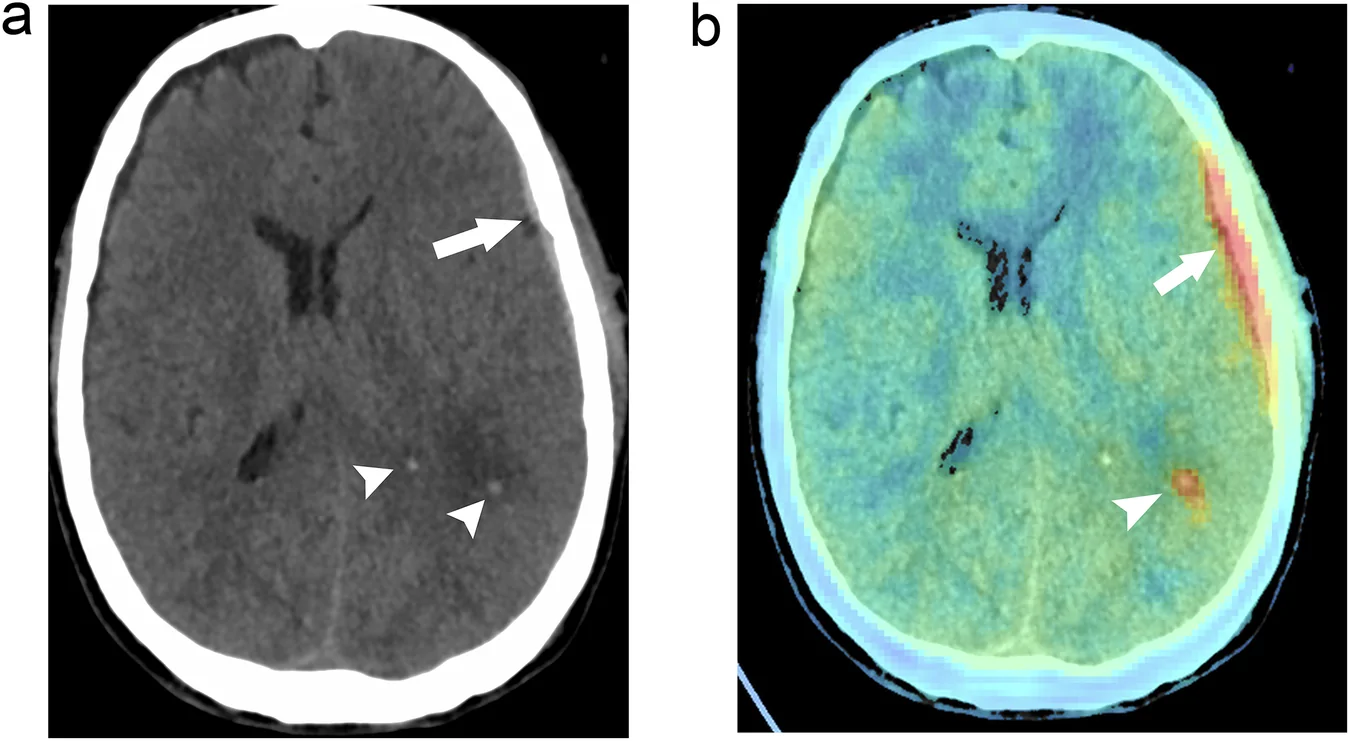
ICH, true-positive for the AI software. **a** Unenhanced cerebral CT: acute left SDH (arrow) and hemorrhagic contusions (arrowheads). **b** The ICHs are correctly color-labeled by AI on the heatmap. ICH, intracranial hemorrhage; SDH, subdural hematoma

The sensitivity of AI for detecting ICH was good for all types of ICH, ranging from 78.6% for subarachnoid hemorrhage (Fig. 2) to 87.9% for intraparenchymal hemorrhage, except for SDH. The sensitivity of radiology residents for detecting ICH was significantly better than those of the AI in all categories of ICH, ranging from 92.9% for subarachnoid hemorrhage to 100% for epidural hemorrhage, except when AI was detecting an association of multiple types of ICH (Fig. 3), and when ICH was localized in multiple sites, where sensitivities for residents (98.5% and 98.4%, respectively) and AI software (97.7% and 95.2%, respectively) were comparable. The sensitivity of AI was lower for detecting subacute (50%) and chronic SDH (25%), while the sensitivity of residents remained high for all SDH types (Table 4).

**Fig. 4:**
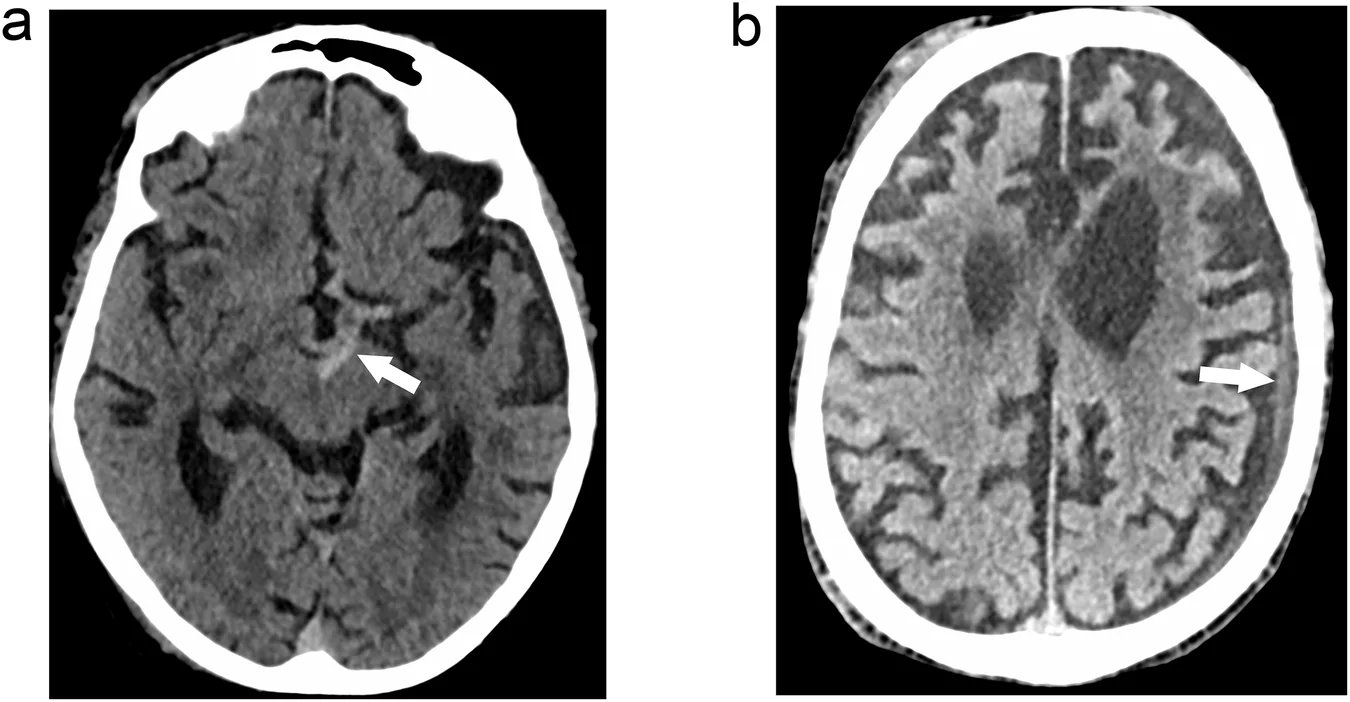
ICH, false-negative for the AI software. **a** Unenhanced cerebral CT: interpeduncular subarachnoid hemorrhage (arrow), not detected by AI. **b** Unenhanced cerebral CT: left subacute SDH (arrow), not detected by AI. ICH, intracranial hemorrhage; SDH, subdural hematoma

**Fig. 5:**
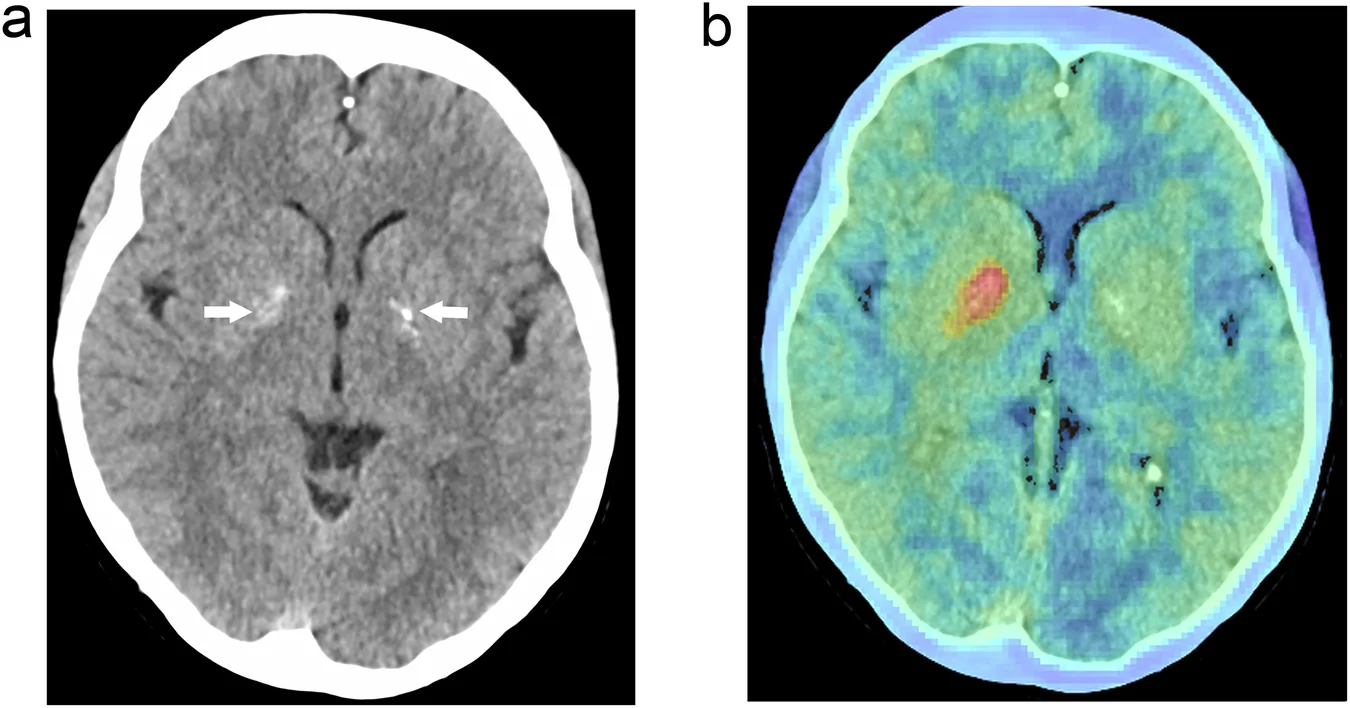
Unenhanced cerebral CT: **a** bilateral basal ganglia calcification (arrows), **b** falsely labeled as ICH on the right side by AI on the heatmap. ICH, intracranial hemorrhage

**Fig. 6:**
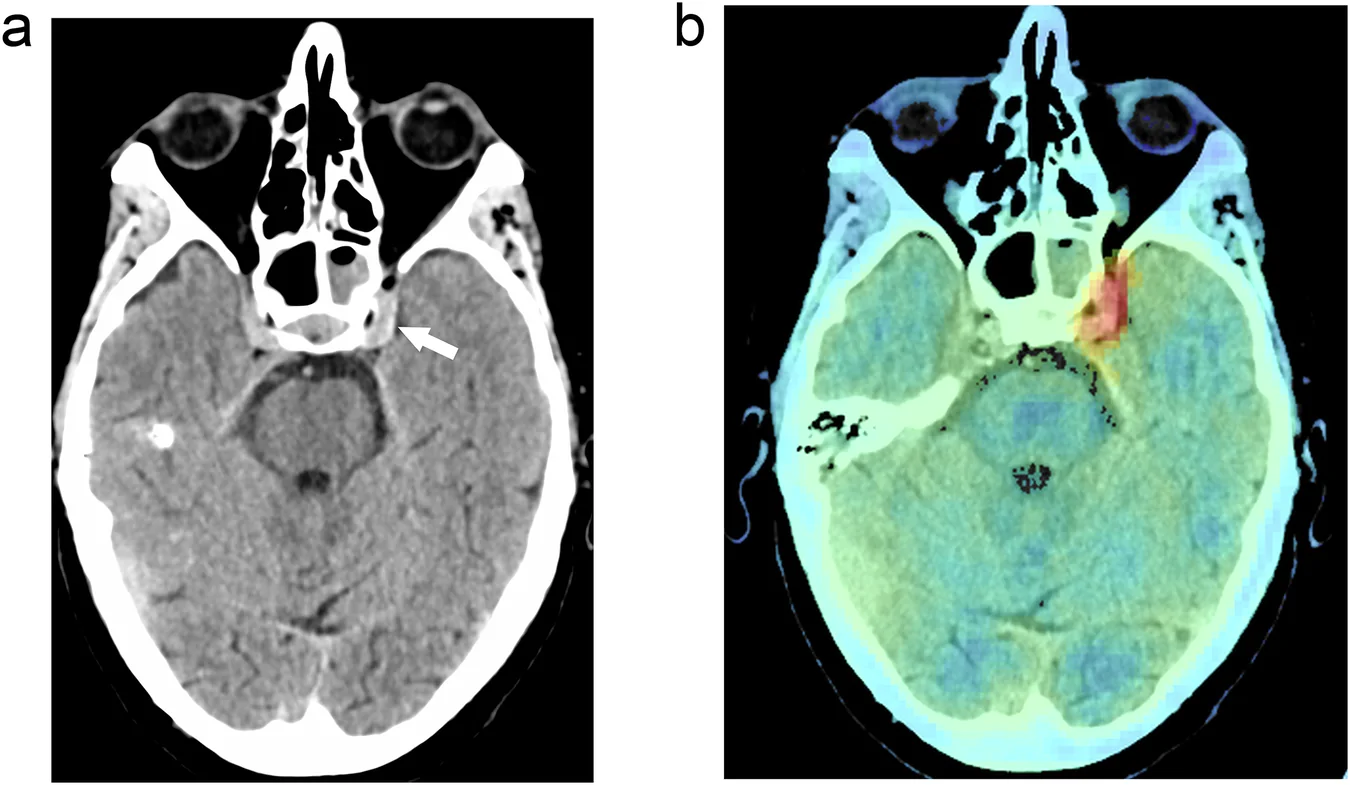
Unenhanced cerebral CT: **a** calcified cavernous segment of the left internal carotid artery (arrow), **b** falsely labeled as ICH by AI on the heatmap. ICH, intracranial hemorrhage

**Table 4:** Sensitivity of the AI software and of the radiology residents by subtype of hemorrhage

	*N* (%)	Sensitivity of AI (%)	*p*-value	Sensitivity of radiology residents (%)	*p*-value	Between-methods *p*-value
Type of ICH (*N* = 331)			< 0.001		0.31	
Subarachnoid	70 (21.1)	78.6		92.9		0.013
Subdural	65 (19.6)	58.5		95.4		< 0.001
Epidural	8 (2.4)	87.5		100		1.00
Intraparenchymal	58 (17.5)	87.9		96.6		0.125
Multiple types	130 (39.3)	97.7		98.5		1.00
Number of sites (*N* = 331)			< 0.001		0.035	
Single	143 (43.2)	69.2		93.7		< 0.001
Multiple sites	188 (56.8)	95.2		98.4		0.11
SDH (*N* = 65)			0.10		0.70	
Acute	53 (81.5)	64.2		94.3		< 0.001
Subacute	4 (6.2)	50.0		100		0.48
Chronic	8 (12.3)	25.0		100		0.013

*ICH* intracranial hemorrhage, *SDH* subdural hematoma

The AI software missed ICH in 53 cases; these consisted of SDH (*n* = 27), focal subarachnoid hemorrhage (*n* = 15) (Fig. 4), small parenchymal hemorrhages (2–5 mm, *n* = 7), association of multiple hemorrhagic types (*n* = 3), and a 4-mm epidural hematoma (*n* = 1). The missed SDH by the AI consisted of acute SDH (2–5 mm thickness, *n* = 19), mainly localized along the falx or tentorium cerebelli, chronic SDH (*n* = 6), and subacute SDH (*n* = 2).

Radiology residents missed ICH in 12 cases; these consisted of SDH (*n* = 3), focal cortical subarachnoid hemorrhage (*n* = 7), residual postoperative ICH (*n* = 1), and a 12 mm parenchymal hemorrhage (*n* = 1). The missed SDH by the resident consisted of acute, 2–5 mm thickness SDH, localized along the tentorium cerebelli (*n* = 2) and along the left convexity (*n* = 1).

Among the twelve cases of ICH falsely labeled as negative by residents, six were labeled positive by the AI. These consisted of focal subarachnoid hemorrhage (*n* = 3), acute SDH (*n* = 1), parenchymal hemorrhage (*n* = 1), and residual postoperative ICH (*n* = 1).

AI software mislabeled 101 negative cases as positive for ICH. They consisted of dural thickening (falx/tentorium cerebelli, *n* = 35), spontaneously dense intracranial masses (*n* = 16), artifacts (*n* = 16), intracranial calcifications (*n* = 10) (Fig. 5), intracranial vessels (*n* = 10) (Fig. 6), and miscellaneous abnormalities (*n* = 14), such as dense cortex or surgical material. No cases labeled positive for ICH by the AI software were missed by the attending radiologists. Among the false-positive AI findings, 51% (52/101) occurred in examinations performed for suspected ICH as the primary indication, 37% (37/101) in studies obtained for suspected ischemia vs ICH, and 12% (12/101) in examinations performed for suspected ICH vs other intracerebral conditions.

## Discussion

Our study compared the diagnostic performance of an AI software, to detect ICH on unenhanced CT scans in the emergency setting and that of radiology residents, obtained from their preliminary CT reports, issued during real-life workflow, to the final report of attending radiologists, in a population of patients imaged for suspected ICH.

In our study, the AI software achieved an overall sensitivity of 84% for detecting ICH, significantly lower than the sensitivity of 96.4% for radiology residents (*p* < 0.001), with a significant difference in specificities (94.4% vs 99.6%, respectively, *p* < 0.001). The AI performance for ICH was comparable to that reported by other centers using the same software, as shown in Table 5. Variations in sensitivity and positive predictive values for the AI software among these studies may be attributed to differences in population characteristics, methodological approaches, and ICH prevalence. Nonetheless, the sensitivity of AI of 84% for detecting ICH in our study falls within the range reported in the current literature [5, 12–15]. Our findings revealed that the AI software achieved the highest sensitivity (97.7%) in cases with the concomitant presence of multiple ICH types and in the presence of ICH in multiple sites. This high diagnostic performance may be attributed to the AI’s intrinsic functioning: the software might label just one of the multiple sites of hemorrhage, and the case automatically becomes positive for ICH, regardless of the ICH location or type. Our results are concordant with the findings of Voter et al and Sayem et al, when evaluating the same AI software [5, 14].

**Table 5:** Comparison between studies, using the same AI software, with regard to diagnostic performance for ICH detection

	Sensitivity (%)	Specificity (%)	Positive predictive value (%)	Negative predictive value (%)
Current survey	84	94.4	73.2	97
Kau et al [13]	68.2	96.8	69.5	96.6
Seyam et al [5]	87.2	93.9	70.5	97.8
Voter et al [14]	92.3	97.7	81.3	99.2
Davis et al [12]	73	94	73	94
Zia et al [15]	82.1	97	64.8	98.8

*ICH* intracranial hemorrhage

We observed a common pattern of missed ICH by AI in unenhanced CT-scans

Firstly, we observed that small hemorrhages were more difficult to detect, regardless of their type and location. Secondly, we noted that hemorrhages situated along the cerebellar tentorium, or the falx, were particularly difficult to identify by the AI. Lastly, the density of the hemorrhage seemed to be a contributing factor: subacute or chronic SDHs, with lower CT densities than acute SDHs, were harder for the AI to detect.

These differences in sensitivity of AI for detecting ICHs, depending on its size, location, and density, are in line with those reported in other studies, conducted in different hospitals, and using CT scanners from various manufacturers [5, 14, 15]. Therefore, these results suggest that the false-negative cases for AI were not related to the type of CT equipment, acquisition mode, or slice thickness, but rather to the intrinsic operating mode of the software, which should be considered when using the AI system.

In our study, the false positive cases of ICH by the AI software were varied, but most frequently due to dural thickening, beam hardening artifacts, and calcifications. The common denominator among these cases was the presence of structures with high densities on non-contrast cerebral CTs.

These types of false-positive cases for the AI software have also been highlighted in other studies [5, 13, 14]. They may be linked to the software’s functioning and to the type of cases on which it was trained. Depending on the clinical indications for CT, the false-positives may lead to increased ED length of stay [16], additional follow-up imaging [17, 18], or inappropriate treatment changes, such as temporary discontinuation of anticoagulation medication [17], or non-administration of intravenous thrombolytic therapy in patients with acute ischemia [19]. However, in daily practice, these images typically would not cause confusion with ICH for radiologists interpreting CT scans, as they are easily recognized as various hyperdense intracranial abnormalities not related to ICH [5, 14].

Our results also showed that the sensitivity of the AI was lower when the primary indication for the CT scan was the detection of ICH, in contrast with the residents, who demonstrated excellent performance regardless of the indication. We do not have a clear explanation for these findings. One possible reason was the difference in the prevalence of positive examinations for ICH across different indication subgroups. Specifically, ICH was more prevalent in the subgroup of patients for whom the indication for CT was suspicion of ICH. This high ICH prevalence may also include cases of subacute or chronic hemorrhages, which are challenging for AI to detect, as also suggested by Zia et al [15], in the analysis of their study group.

Our study showed that, in our center, residents outperformed the AI software in detecting ICH, regardless of the type and the site of ICH, with a low rate of discrepancies between attendings’ final reports and residents’ preliminary reading. This excellent diagnostic performance of radiology residents may reflect the effectiveness of their preparation for emergency radiology in our department, as they start to autonomously deliver preliminary reports for emergency CT examinations at the end of their 2nd year of residency, after at least one year of mandatory supervised training in body and neuro CT. These current results are concordant with those obtained from a previous quality assessment of our emergency radiology activity [20], which revealed a 3% rate of discrepancies between residents and attendings when reporting for emergency neurological CT examinations.

To the best of our knowledge, only one previous study, by Kau et al, reported the diagnostic performance of radiology residents when compared to the same AI software [13]; however, their methodology was different from ours, in that residents’ interpretation was obtained retrospectively in simulated, “under pressure” conditions and not during real life reporting, like in our study. Nonetheless, their results were concordant with ours: in their evaluation, residents achieved a 94.9% sensitivity when compared to 68.2% sensitivity of AI for ICH detection [13]. These results suggest that radiology residents still outperform AI in detecting ICH on unenhanced CTs, indicating limited utility for implementing AI in real-life clinical settings at this stage.

We must acknowledge some limitations of our study. First, the reference standard used was the attending radiologist’s final report, and no further follow-up was conducted for cases with discrepancies between the attending and the AI. However, in daily clinical practice, the attending’s final report serves as the basis for patients’ management, and this was the justification for considering it as the reference standard. Second, inter-rater variability was not assessed, as the purpose of our study was to evaluate real-world data and not differences between individual residents. Third, we did not stratify the analysis by CT-scanner type, because this approach is consistent with our clinical workflow. Moreover, the AI software is FDA and CE-approved, without CT-scanner-specific restrictions [9, 10], and previous studies reported no differences in performance across multiple CT systems [14]. Fourth, the small number of cases in some ICH subgroups, particularly epidural hematoma and subacute/chronic SDH, may yield limited statistical power. Thus, statistical significance among these subgroups should be interpreted with caution. Nevertheless, the findings remain clinically relevant, as they provide descriptive insights into AI performance in clinically important hemorrhage subtypes. Finally, this analysis used the version of the software available at the time of the study; thus, we cannot assert that future versions of the same software would not yield different results.

In conclusion, our study showed that radiology residents, autonomously interpreting emergency CT examinations in patients with suspected ICH, had a better performance in detecting ICH compared to the AI software. The AI software demonstrated a good diagnostic performance in identifying certain subgroups of ICH, particularly in the presence of multiple hemorrhagic sites or associations of multiple hemorrhagic types.

## Abbreviations

AI: Artificial intelligence
ED: Emergency department
ICH: Intracranial hemorrhage
PACS: Picture archiving and communication system
SDH: Subdural hematoma
